# SynesthesiaColorPicker: An open-source color picker for online synesthesia research

**DOI:** 10.3758/s13428-025-02882-1

**Published:** 2026-03-04

**Authors:** Nicholas Root

**Affiliations:** Brain and Cognition, Department of Psychology, University of Amsterdam, 1018 WS Amsterdam, Netherlands

**Keywords:** Synesthesia, Test–retest consistency, Grapheme–color, Color picker

## Abstract

Synesthesia is a neurological phenomenon in which healthy individuals experience additional, automatic, and consistent perceptions unrelated to veridical sensory input. For most (but not all) synesthetes, this additional experience is a *color*: for example, grapheme–color synesthetes experience colors for letters of the alphabet. Measuring these color associations is of central importance to synesthesia research, but there is no standard color picker “tool” that researchers can adapt to use in their own experiments: each researcher must code their own. This is a barrier to entry for synesthesia research, and additionally creates potential methodological confounds because different researchers make color pickers with different properties. SynesthesiaColorPicker is an open-source, mobile-friendly color picker tool that can be integrated with two popular online experiment platforms (Qualtrics and lab.js/Open Lab) without any prior programming knowledge. The templates, underlying JavaScript code, and detailed instructions are available for download on a GitHub repository. Furthermore, a comparison between data collected with SynesthesiaColorPicker and with the Synesthesia Battery shows that two methodological design choices in SynesthesiaColorPicker overcome measurable confounds in existing color picker methodology.

## Introduction

Synesthesia is a neurological phenomenon in which healthy individuals experience additional, automatic, and consistent perceptions. For example, grapheme–color synesthetes experience linguistic symbols as having a consistent color (e.g., “J is dark purple”). Until relatively recently, synesthesia’s status as a genuine perceptual phenomenon was debated (Blake et al., 2005). A key advance in the study of synesthesia was the development of the *test–retest consistency* measure of synesthesia: synesthetes are asked to describe their experiences, are given a surprise retest, and their consistency over time is quantified (for a review, see Johnson et al., 2013). For example, grapheme–color synesthetes will easily choose the same color for a particular grapheme across months or even years, whereas controls find such a task to be nearly impossible, because they must try to use mnemonic strategies rather than simply reporting conscious perception (Asher et al., 2006). Test–retest consistency is now considered the “gold standard” diagnostic tool for synesthesia, and few studies of synesthesia are published without first verifying their participants’ synesthesia using a test–retest measure (but see Root et al., 2025).

Although there are more than 50 known types of synesthesia (Day, 2022), synesthesia of the type X→color (grapheme–color, music–color, etc.) is by far the most common (~95% of all synesthetes; Simner et al., 2006). For this reason, test–retest questionnaires frequently employ *color pickers*: synesthetes are presented a stimulus (e.g., a grapheme) and must use a color picker to indicate which synesthetic color they experience. Color picker data are useful beyond just measuring test–retest consistency. For example, synesthetes’ color associations are not random: *certain* letters tend to be associated with *certain* colors (e.g., Rich et al., 2005; Simner et al., 2005), and synesthetic colors have been shown to be influenced by visual (e.g., Brang et al., 2011; Watson et al., 2012), semantic (e.g., Mankin & Simner, 2017), and cultural (e.g., Root et al., 2019) factors. Furthermore, some experimental tasks require stimuli customized to each synesthete: for example, synesthetic Stroop tasks (e.g., Dixon et al., 2000) require presentation of stimuli that are congruent (or not) with a synesthete’s specific color associations.

Measuring color associations is therefore of central importance for synesthesia research. The popular Synesthesia Battery (Eagleman et al., 2007) enables researchers to collect test–retest consistency data for a few types of synesthesia. However, it does not allow researchers to customize the test (e.g., add different stimuli, answer options, or synesthesia types). Furthermore, the Synesthesia Battery requires participants to share their personal information and data with external researchers, which may be incompatible with European data privacy laws and with the research ethics guidelines at many universities.

Unfortunately, programming expertise (JavaScript, Python, etc.) is a prerequisite for integrating a color picker into experiments (e.g., Cuskley et al., 2019). Popular experiment “builders” such as Qualtrics, lab.js, and PsychoPy do not include “drag-and-drop” color picker components. This presents a barrier to entry for synesthesia research, and it has also created a fractured methodological environment. Many researchers have created their own color pickers, and as a result, the color picker tasks in synesthesia studies are often quite different from each other. For example, the Synesthesia Battery (Eagleman et al., 2007) uses an HSL color picker in which all three HSL dimensions (hue, saturation, and lightness) can be manipulated. In contrast, Smilek et al. (2007) use a similar HSL color picker, but only allow participants to select hue and brightness (saturation is always at its maximum). Some studies (e.g., Asher et al., 2006; Simner et al., 2009) do not use an adjustable color “picker,” instead presenting participants with square color patches to choose from. Other studies (e.g., Simner et al., 2005; Rouw et al., 2014) collect verbal descriptions of the color experience, which are then converted to color categories by human coders.

Synesthetes do not experience colors for every letter (Cytowic & Cytowic, 1989), and synesthesia studies also vary in how they handle such “no color” trials. For example, the Synesthesia Battery allows synesthetes to indicate that they experience “no color” (in which case they “skip” the trial). In contrast, in Asano and Yokosawa’s (2011) color picker task, synesthetes were instructed to choose black if a presented grapheme did not elicit a synesthetic color (potentially confounding because synesthetes can experience black associations; Melero et al., 2014). In addition, the “no color” option does not make sense when measuring implicit grapheme–color associations in non-synesthetes: since they do not experience colors for letters, they would presumably answer “no color” every trial. In these experiments, non-synesthete controls are often explicitly instructed that they cannot choose the “no color” option (e.g., Rothen et al., 2013; Asano & Yokosawa, 2011). However, in these studies, non-synesthetes can still “skip” trials by leaving the color picker on its randomly initialized value. Importantly, the Synesthesia Battery does not include in its output the (randomized) starting value of each trial, so it is not possible for experimenters to detect such “skipped” trials.

It is not implausible that these methodological choices could have an impact on experimental results. For example, Simner et al. (2006) measured colors using verbal elicitation and found that non-synesthete controls (vs. synesthetes) chose colors in proportion to their ease of (verbal) generation (Battig & Montague, 1969). In contrast, Rouw and Root (2019) measured colors using a color picker and found that non-synesthete controls (vs. synesthetes) chose colors in proportion to their size (percentage of pixels in the color category) in HSL space. Taken together, these results suggest that the method by which color is measured might influence the colors that are ultimately chosen, especially in non-synesthetes, who are reporting an implicit association rather than conscious perception.

In the present work, I present SynesthesiaColorPicker, an open-source color picker for synesthesia research that can be implemented in popular online experiment platforms and can be used without any authorship or data sharing obligations. The design of SynesthesiaColorPicker differs in two key ways from the popular Synesthesia Battery: it uses a hue wheel rather than a hue slider, and it does not allow color picker trials to be skipped. The experiments described here use data collected from the Synesthesia Battery and SynesthesiaColorPicker to demonstrate that these methodological differences can influence the responses of synesthetes and non-synesthete controls.

## SynesthesiaColorPicker: An open-source web-based color picker for synesthesia experiments

SynesthesiaColorPicker is an online color picker that is designed to function on any website that supports JavaScript. With attribution (i.e., by citing this paper), it can be freely used, modified, and distributed, enabling any researcher to create their own test–retest consistency and color picker tasks, with no authorship or data sharing obligations. The templates and code can be downloaded from GitHub: https://github.com/nicholasbroot/synesthesiacolorpicker. The easiest way to implement SynesthesiaColorPicker for your own research is to download one of the pre-made consistency test templates for Qualtrics or Open Lab. These templates replicate the function of the Synesthesia Battery and require no programming knowledge to use. The GitHub page includes detailed instructions for how to set up these templates, and sample R code for how to analyze the data they produce. Below is a short summary of the color picker design, use, and output, as well as several examples of how it can be modified and extended.

## SynesthesiaColorPicker: Design and use

SynesthesiaColorPicker is a jQuery and HTML Canvas-based color picker designed to integrate into the Qualtrics and lab.js platforms. The color picker code is adapted from Wittens (2017), updated to reflect jQuery and browser changes, and modified for experimental use (e.g., randomizing the orientation of the hue wheel on each initialization). The Qualtrics and lab.js code are written by author NR. All code is free to use and modify with attribution.

**Fig. 1:**
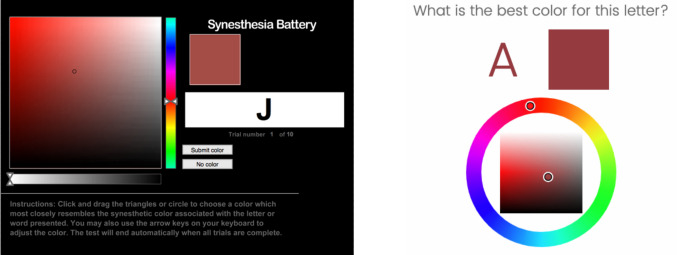
An example color picker trial in the Synesthesia Battery (left panel) and the SynesthesiaColorPicker (right panel). In the Synesthesia Battery trial (left panel), the endpoint hue of the hue slider is pure cyan (HSL hue angle 180°)

The color picker contains two parts (Fig. 1, right): a circular hue “wheel” that allows participants to select the hue component of HSL (hue, saturation, lightness), and a rectangular “palette” that allows participants to select the saturation and lightness. Participants can interact with the color picker by clicking or by clicking and dragging. In either case, each change of the selected color calls the JavaScript function colorChange(), to which any number of behaviors can be added. In the fully functional test–retest consistency template (requiring no coding to run), this function is set up to automatically adjust the color of the letter on the screen as well as the rectangular color swatch (Fig. 1, right).

## SynesthesiaColorPicker: Test–retest consistency template

The GitHub repository includes a working test–retest consistency template for both Qualtrics and lab.js, along with detailed step-by-step instructions for how to create a fully working experiment using the template. This replicates the core functionality of the Synesthesia Battery: On each trial, a participant first reports their subjective experience (indicating they experience no color, one color, or multiple colors for a letter). If they report experiencing multiple colors for a letter, they then complete an additional free-response question asking them to describe their experience. Finally, they are directed to the color picker (regardless of their response—even if they indicate experiencing no color) and are instructed to choose the “best” color for the letter. This process continues for each letter (in random order). After choosing colors for all letters in the alphabet, there is a mandatory 10-second break, after which the process repeats twice. On the second and third repeat, only the color picker question is presented (participants indicate their subjective experience only on the first trial). At the end of the experiment, they see a debriefing text and are then optionally redirected to a credit-granting website. The template includes blank sections for consent form, instructions, and debriefing text, and the GitHub page includes detailed step-by-step instructions for how to distribute the survey and how to redirect (e.g., to automatically grant research credits on the commonly used SONA platform).

### Data format and preprocessing

Both the Qualtrics and lab.js test–retest consistency templates yield experiments that output a significant amount of data. The default format of the data is not straightforward to clean (especially for Qualtrics), and contains significant amounts of extraneous information. To assist researchers in processing data from these templates, the GitHub repository includes preprocessing scripts, written in R, to process both the Qualtrics and the lab.js output. These preprocessing scripts transform the data into a standard long-format data frame. The scripts also include additional sample code snippets that demonstrate how to calculate test–retest consistency, how to exclude participants based on response features, and how to visualize the color picker data using the *ggplot2* R package.

## SynesthesiaColorPicker: Modifications and extensions

### Modifying the existing color picker

Even for users without programming knowledge, it is straightforward to modify the GitHub templates to customize the page layout, add questions/questionnaires, and change the stimuli that are tested (e.g., use a different alphabet). There are several aspects of the color picker that are straightforward to modify, requiring no previous coding experience. Changing the font and font size, changing the size of the color picker, and changing the background color of the page can be accomplished in Qualtrics using menu functions, and in lab.js by modifying the value of a clearly marked variable. The GitHub page describes in step-by-step detail how to accomplish these cosmetic changes.

### Extending the features of the color picker

For users with programming knowledge, SynesthesiaColorPicker can also be modified in more complex ways. Below are three examples of how SynesthesiaColorPicker can be extended based on a specific use case for the color picker.

#### Example use case: “Piping” color values forward

 A simple but powerful feature of the Qualtrics implementation of SynesthesiaColorPicker is the ability to “pipe” answers into subsequent questions. This feature is built into the Qualtrics GUI, and is accessed via a dropdown menu in the question design. An additional template on GitHub, qualtrics_template_piping_example.qsf, demonstrates how this feature can be used. After collecting test–retest data, the experiment then presents the participant with a letter that is colored either correctly or incorrectly, and prompts them to indicate whether the letter is in the correct color. This feature can also be implemented in lab.js, but requires knowledge of JavaScript (lab.js data format: https://labjs.readthedocs.io/en/latest/reference/data.html).

#### Example use case: Dynamic background color

 When participants use the default color picker to choose white, the letter and color swatch disappear because they are identical to the background color of the page. In my own experiments, I keep the background color white throughout the experiment to avoid potential confounds of color contrast effects. However, it is plausible that in some experiments it is more important that the participant always be able to see the letter on the screen when they choose white. One method to address this is to set the background color of the divs containing the letter and color swatch to black when the participant chooses a particularly bright color. An additional template on GitHub, qualtrics_template_bgchange_example.qsf, demonstrates how this feature can be added.

#### Example use case: Color “painting”

 Some synesthetes, especially in nonalphabetic languages, report experiencing graphemes as multicolored (Root et al., 2020; Root et al., 2025). In the default version of the test–retest consistency experiment, these experiences are accommodated using a free-response text box in which the participant is asked to describe their experience. An alternative is to allow the participant to “paint” the colors they experience, and analyze synesthetic colors on a pixel-by-pixel level within each grapheme (Root et al., 2025; Zhang et al., Submitted). An additional template on GitHub, Qualtrics_template_painter_only.qsf, demonstrates how this feature can be implemented. This is a significant extension to the base of SynesthesiaColorPicker, and implementing this template as an experiment requires significantly more knowledge of JavaScript than the other examples. The template demonstrates how many features can be added: live interaction with an additional HTML Canvas, saving more complicated output via an additional response box, importing functions from an additional JavaScript file, etc.

## SynesthesiaColorPicker versus Synesthesia Battery

SynesthesiaColorPicker is similar in function and design to the popular Synesthesia Battery; indeed, it was first designed to be a direct replacement for it. However, the color pickers differ in two key ways. First, SynesthesiaColorPicker uses a circular hue wheel rather than a linear hue slider (Fig. 1, left vs. right panel). Second, SynesthesiaColorPicker separates the subjective report of experiencing a color (or not) from the act of choosing a color: on each trial, participants are first asked how many (if any) colors they experience for the stimulus, and then, *regardless of their answer*, they choose the “best” color for the stimulus using the color picker. Study 1 and Study 2 demonstrate why these methodological design choices are advantageous.

## Study 1: The influence of color picker design on color association data

Many studies have used the Synesthesia Battery (Eagleman et al., 2007) to measure test–retest consistency in grapheme–color synesthetes. SynesthesiaColorPicker was designed to be as similar as possible to the Synesthesia Battery, but open-source and modifiable. One difference between the color pickers is that SynesthesiaColorPicker was designed with a circular hue picker (Wittens, 2017) with no “endpoints” (Fig. 1, right), in contrast to the original Synesthesia Battery’s linear hue picker with two endpoints (Fig. 1, left). We initially believed this difference to be cosmetic and inconsequential, but later discovered a quirk of the linear hue picker of the Synesthesia battery that might set it apart from the circular picker: The endpoints of the hue picker were randomized to pure red, yellow, green, cyan, blue, or magenta (i.e., HSL hue angles of 0, 60, 120, 180, 240, or 300).^1^ As a result, if participants chose a color by dragging their mouse to the endpoint of the hue picker, there could conceivably be a bias in the data, making it look as if participants picked very “pure” red, green, blue, etc., more often than would be expected. Indeed, using Synesthesia Battery data, we previously found that participants in general (and synesthetes in particular) chose pure hues more often than expected (Rouw & Root, 2019). At the time, we attributed this effect to psychology (tending to associate graphemes with “pure” or “basic” colors), and did not consider the possibility that this could have instead been an artifact of the color picker design.

Here, I use SynesthesiaColorPicker’s circular hue wheel to test whether the “endpoint” colors of a linear color picker influence behavior on the color picker task—specifically, whether participants are more likely than chance to choose an exact endpoint color, because they drag their mouse to the edge of the picker. If participants are influenced by the existence of endpoints in the Synesthesia Battery color picker, colors with the exact hue angles of Synesthesia Battery endpoints (0, 60, 120, 180, 240, 300) should be chosen more often in the Synesthesia Battery data than in the SynesthesiaColorPicker data.

## Methods

### Previous dataset (Synesthesia Battery data)

The Synesthesia Battery dataset is the publicly available data from native speakers of Dutch reported in Rouw and Root (2019). This dataset included 163 participants (130 female, 21 male, 12 not reported; age data were not collected). Further details about the participant sample, methods, and procedure can be found in Experiment 1 of Rouw and Root (2019).

### Previous Dataset (SynesthesiaColorPicker data)

The SynesthesiaColorPicker dataset is the data from native speakers of Dutch in Root et al. (2025). In this dataset, there were 202 participants (78% female; age mean/*SD*, 29.7 ± 13.0). Further details about the participant sample, methods, and procedure can be found in Experiment 1 of Root et al. (2025).

### Data preprocessing (both Synesthesia Battery and SynesthesiaColorPicker data)

The Synesthesia Battery data had already been preprocessed (Rouw & Root, 2019) as follows: first, participants were excluded who reported “no color” for at least 50% of letters; second, participants were excluded who chose black on more than 80% of trials (suggesting they misunderstood the task, and chose the printed letter color); third, participants were excluded who admitted after the experiment that they did not follow task instructions. Only the first trial for each letter was included in the final dataset (synesthetes’ and non-synesthetes’ color choices can be directly compared on their first answer, but not on subsequent ones, because synesthetes will simply report their color again, whereas there may be large individual differences in the strategies non-synesthetes use on retest). Finally, for the present work, only the subset of responses that were from native Dutch speakers was included, in order to better match this dataset with the SynesthesiaColorPicker data. The final Synesthesia Battery dataset consisted of 163 participants: 126 participants were synesthetes, and 37 were non-synesthetes.

The SynesthesiaColorPicker data were preprocessed using the same steps as in Rouw and Root (2019). Although the SynesthesiaColorPicker data included self-report (which we argue is a better measure of synesthesia; Root et al., 2025), these data were not used because they were not collected in Rouw and Root (2019). Instead, synesthesia was operationalized as in Rouw and Root (2019): test–retest consistency in the CIELuv color space less than or equal to 135 (Rothen et al., 2013). Furthermore, as in Rouw and Root (2019), participants were excluded if they chose black (as operationalized using the Colournamer algorithm of Mylonas et al., 2013) on more than 80% of trials (three participants excluded), or if they indicated that they did not experience a color for more than 50% of graphemes (10 participants excluded). As in Rouw & Root (2019), only the first trial for each letter was retained for further analysis (the second and third trials were only used to calculate test–retest consistency). Finally, in both the Synesthesia Battery and SynesthesiaColorPicker datasets, achromatic trials (the participant chose white, gray, or black, as operationalized by the Colournamer algorithm of Mylonas et al., 2013) were excluded. The hue angle of achromatic colors is undefined, so the present hypothesis does not make a prediction about these trials; two participants chose only achromatic colors and were thus excluded. The final SynesthesiaColorPicker dataset consisted of 187 participants: 94 synesthetes and 93 non-synesthetes.

## Results

**Fig. 2:**
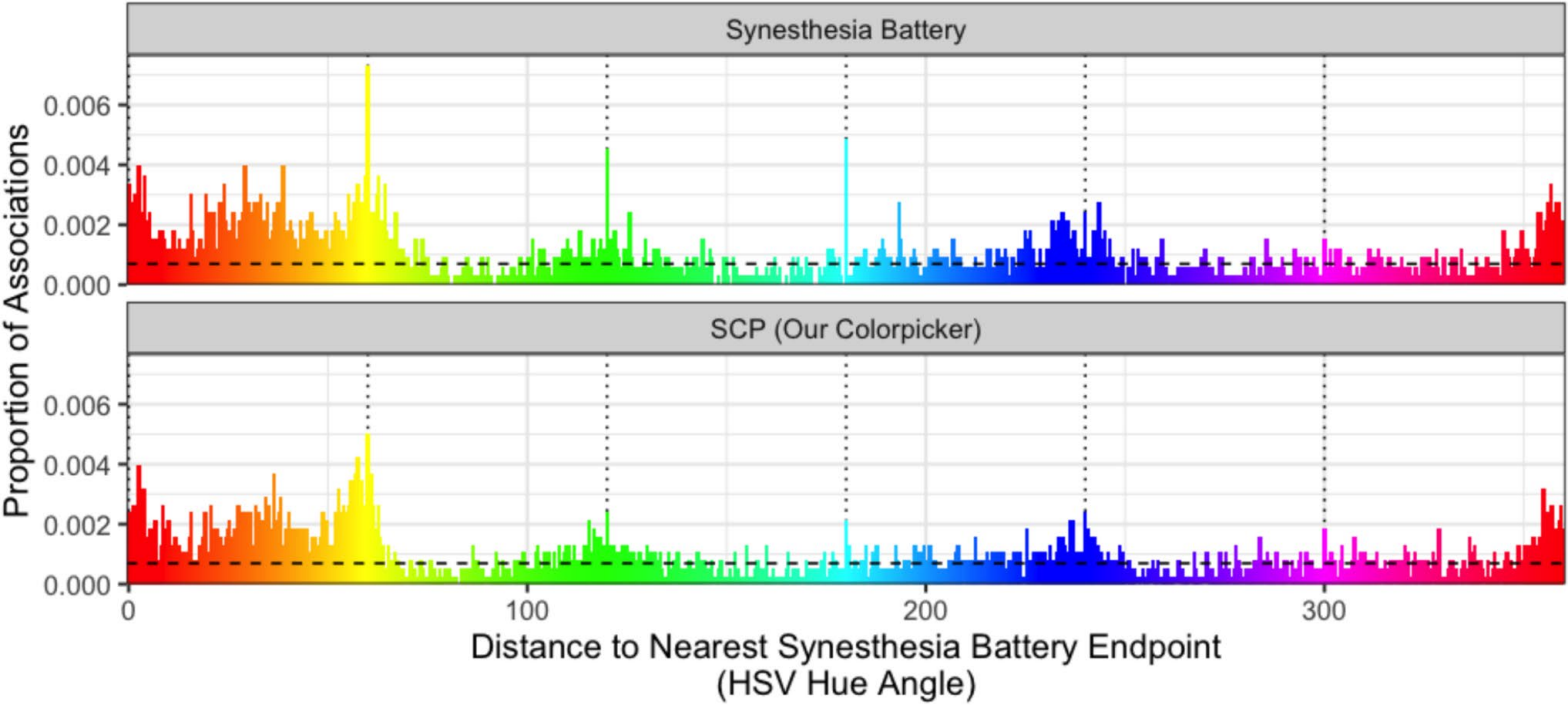
The proportion of associations as a function of their distance to the nearest endpoint on the Synesthesia Battery, for Synesthesia Battery data (top panel) and SynesthesiaColorPicker data (bottom panel). Bars have a binwidth of 1/4 degree. The horizontal dashed line is the proportion expected by chance (i.e., $(\frac{1}{4})(\frac{1}{360})$). The vertical black dotted lines indicate the Synesthesia Battery hue endpoints (0, 60, 120, 180, 240, 300). Note the clearly visible “spikes” at the endpoints in the Synesthesia Battery data (especially for yellow, green, and cyan), indicating that participants are particularly likely to choose hues at the exact endpoints of the hue picker.

Figure 2 depicts the distribution of associations as a function of distance to the nearest Synesthesia Battery endpoint, for Synesthesia Battery and SynesthesiaColorPicker data. The leftmost bar indicates the proportion of associations at the exact endpoint hues (exact HSL hue angle 0, 60, 120, 180, 240, or 300). Qualitatively, this bar is noticeably larger in the Synesthesia Battery data—consistent with the color picker design influencing behavior. Indeed, endpoint colors were chosen significantly more often in the Synesthesia Battery dataset than in the SynesthesiaColorPicker dataset (${\chi }^{2}\left(1\right)=5.15, p=0.023, \phi =0.027$). The proportion of endpoint colors in the SynesthesiaColorPicker dataset (1.62%) is not significantly different from the 1.67% expected by chance (binomial test; $p=0.90, 95\%\text{ CI }[1.24, 2.08]$). In contrast, the percentage of endpoint colors in the Synesthesia Battery dataset ($2.4\%$) is significantly higher than would be expected by chance (binomial test; $p=0.002, 95\%\text{ CI }[1.91, 2.99]$).

The distribution of color associations collected with the Synesthesia Battery and SynesthesiaColorPicker differs *overall*, but does it also differ in synesthetes versus non-synesthetes (conceptually replicating the analysis of Rouw & Root, 2019)? In the Synesthesia Battery dataset, synesthetes were more likely than non-synesthetes to choose colors at the exact endpoints (2.73% vs. 1.44%; ${\chi }^{2}\left(1\right)=3.90, p=0.048, \phi =0.035$). We first reported a similar result in Rouw and Root (2019), although it was operationalized differently (“within one degree of hue angle of unique red, yellow, green, or blue” rather than “exactly 0, 60, 180, 240, or 300 degrees HSL hue angle”) because at the time we were not aware of the existence of specific endpoint colors in the Synesthesia Battery; we instead interpreted the effect as the likelihood of choosing “pure” or “unique” hues (Miyahara, 2003). However, the present data strongly suggest that the result in Rouw and Root (2019) was an artifact of the Synesthesia Battery color picker design: in the SynesthesiaColorPicker dataset, synesthetes and non-synesthetes chose endpoint colors at statistically comparable rates (1.52% vs. 1.74%, respectively; ${\chi }^{2}\left(1\right)=0.15, p=0.70, \phi <0.01$).

## Discussion

A very subtle methodological difference in color picker design—whether or not certain hues are consistently at the “edge” of a color picker—can measurably influence the colors that are reported in a synesthesia experiment. These results are consistent with the hypothesis that participants select the colors at the endpoints of a hue slider more often than when those colors are not at the endpoints. The SynesthesiaColorPicker does not have this issue: there are no endpoints on the circular hue wheel, and its orientation is completely random on each trial.

This “endpoint” confound plausibly influenced one of the results reported in Rouw and Root (2019). We originally reported that synesthetes chose unique hues (“pure” red/green/yellow/blue) more often than non-synesthetes, but the present results suggest an alternate interpretation: synesthetes were more likely to use the endpoints of the hue slider to choose their unique color associations. It is surprising that synesthetes are more prone to this behavior than non-synesthetes; anecdotally, we find that synesthetes tend to be much more careful and take much more time than controls to choose the exact color they want. One speculative explanation for these results is that synesthetes are more likely to notice or care that the edge of the hue picker had been set to a unique hue by the experimenters and thus made a conscious cognitive decision to drag the hue slider to its edge to obtain the “reddest red,” the “yellowest yellow,” and so on. Importantly, the key result of Rouw and Root (2019)—a synesthetic “palette” in which synesthetes are more likely to choose warmer colors—is successfully replicated in data collected using the SynesthesiaColorPicker (Root et al. 2025); it is only the “unique hues” result that is confounded by methodology.

In the newest version of the Synesthesia Battery, the endpoints of the hue slider are now set to a completely random hue. This is a substantial methodological improvement over the original design and should prevent the appearance of an “overrepresentation” of unique hues as was observed in Rouw and Root’s (2019) Synesthesia Battery data. However, it is still possible that participants are more likely than chance to drag the hue slider to its edge when choosing a color—the difference is that in the new version, this will be a random color. SynesthesiaColorPicker’s circular hue slider, in contrast, does not have an “edge” to which the mouse can be dragged.

In sum, there is evidence that at least one subtle design choice (the randomization scheme for “endpoints” of a hue picker) can influence synesthesia data in measurable ways, and the design of SynesthesiaColorPicker controls for this confound. The next study tests whether a second design choice also influences synesthesia data: the ability (or not) to skip trials.

## Study 2: The influence of skipped trials on test–retest consistency measures

If control participants are inattentive or uninvested in a test–retest consistency task, they may “skip” trials by choosing the initial (random) color of the color picker. Because these control subjects are *particularly* inconsistent, a threshold based on their data might be too liberal a criterion for synesthesia diagnosis, and may misdiagnose attentive non-synesthetes (many of whom will choose *some* consistent color associations, based on memory or implicit associations; Cuskley et al., 2019; Simner et al., 2005) as true synesthetes. Importantly, the most commonly used color picker tool (the Eagleman Synesthesia Battery) does not record the randomized initial color, making it impossible to determine whether or not a trial response is effortful.

In contrast, synesthetes are often *instructed* to press a button for “no color” when they do not experience a grapheme as having a color. Although this makes logical sense (after all, synesthetes report not experiencing a color), this adds a potential confound to direct comparisons between synesthetes and non-synesthete controls, because we do not know how consistently the synesthete would choose colors for skipped trials. Furthermore, it is an interesting (and unexplored) question in its own right whether synesthetes choose consistent colors for graphemes they do not consciously experience as colored.

In the SynesthesiaColorPicker data, the report of phenomenology (“I experience a color” vs. “I do not experience a color”) was separated from the color picker task: all participants were required to choose a color for every letter. Furthermore, SynesthesiaColorPicker records the (randomly generated) starting color on each trial. Here, I use these data to test whether these methodological choices matter. First, how are the data influenced by participants’ choice to “skip” (or not) trials by leaving the color picker on its random starting value? Second, is there additional information to be gained by forcing synesthetes to choose colors for letters for which they do not have a conscious experience of color?

## Methods

Participants, Methods, and Procedure are identical to the “SynesthesiaColorPicker” sections of Study 1.

***Data preprocessing***. As in Study 1, the Colournamer algorithm of Mylonas et al. () was used to exclude participants who chose black on more than 80% of trials (three participants excluded). Unlike Study 1, synesthetic participants were not excluded on the basis of their “no color” responses (since number of skipped trials is an independent variable in the analysis). Also unlike Study 1, synesthesia was not operationalized using consistency (since consistency is a dependent variable in the analysis) but instead using their answer on the self-report question (indicating “Yes” or “No” to the statement “Letters have a color (e.g., ‘J is yellow’)”; participants answering “Maybe” were excluded—44 participants excluded). The final dataset consisted of 155 participants; 113 participants indicated they had grapheme–color synesthesia, and 42 participants indicated that they did not have grapheme–color synesthesia.

## Results

### Proportion of skipped trials

**Fig. 3:**
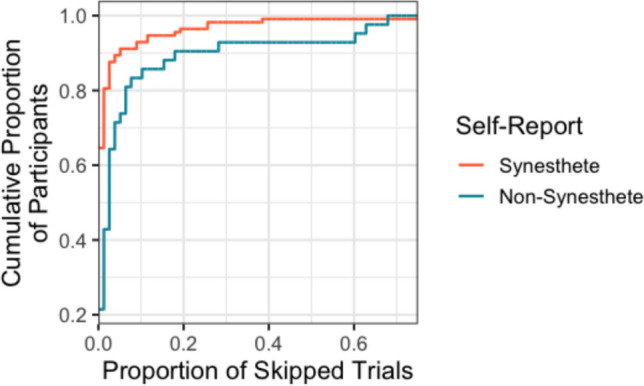
Cumulative distribution of participants as a function of the proportion of skipped trials. Line color depicts self-report of synesthesia. To interpret the graph, observe that, for example, the blue line intersects the point $x=0.2,y=0.9$; this means that 90% of non-synesthetes skipped fewer than 20% of trials, or equivalently that 10% of non-synesthetes skipped at least 20% of all trials.

A “skipped trial” was operationalized as a trial in which the color picker was not adjusted, i.e., the final color choice was identical to the randomly generated initial color. Figure 3 depicts the cumulative distribution function for skipped trials, separated by subjective report of experiencing synesthesia (yes, no, maybe). Non-synesthete controls frequently skipped trials: approximately 10% of non-synesthetes skipped more than 20% of all trials!

This has real implications for distinguishing synesthetes and non-synesthetes using consistency-based measures. Since skipped trials will (logically) make the participant appear inconsistent, and since synesthetes are generally more consistent than non-synesthetes, synesthetes’ skipped trials will impact their consistency score more than non-synesthetes’ skipped trials, bringing their distributions closer together. For this reason, threshold-based classification (e.g., Rothen et al., 2013) may be more precise when skipped trials are excluded. To quantify this, I replicated Rothen et al.’s (2013) receiver operating characteristics (ROC) analysis, calculating the sensitivity and specificity of consistency score-based classification at the ideal threshold (maximizing sensitivity and specificity). Specifically, sensitivity and specificity were calculated in the full dataset and in the dataset in which skipped trials were excluded. Removing skipped trials improved sensitivity (85.8% vs. 89.3%) without a concomitant decrease in specificity (88.1% for both). In our sample, this corresponds to correctly detecting an additional three synesthetes with no penalty. In sum, “skipped” trials have a measurable impact on synesthesia data, which can be controlled for by measuring the random color to which the color picker is initialized.

### Synesthetes’ responses to “no color” trials

As would be (tautologically) expected, synesthetes reported relatively few (~12%) letters as having no color. To further examine these “no color” responses, a linear mixed effect regression was fit to data from all self-reported synesthetes, with the dependent variable of consistency, independent variable of “no color” (vs. one or more colors), and random effect of participant.

**Fig. 4:**
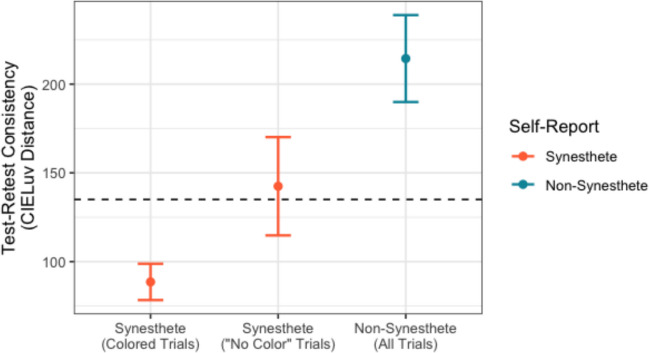
Average test–retest consistency of synesthetes’ colored trials (leftmost bar), synesthetes’ “no color” trials (middle bar), and non-synesthetes (rightmost bar). Color associations (and thus test–retest consistency) for synesthetes’ “no color” trials (middle bar) are not typically collected in most synesthesia experiments. The dotted line (*y* = 135.3) reflects the test–retest consistency threshold which optimally separates English-speaking synesthetes and non-synesthetes (Rothen et al., 2013; but see Root et al., 2025).

As might be expected, synesthetes were less consistent over time for letters with “no color” (likelihood ratio test; ${\chi }^{2}\left(1\right)=50.77, p<0.001,\Delta AIC=48.77$). However, synesthetes’ “no color” letters were still surprisingly consistent: test–retest consistency for “no color” letters was not significantly worse than the 135.3 classic threshold for diagnosing synesthesia (Fig. 4; estimated marginal means; $Z=0.49,p=0.62;95\%\text{ CI }[116.80, 146.39]$).

The surprising consistency of synesthetes’ “no color” trials suggests that synesthetes and non-synesthetes might differ even on only “no color” trials (i.e., removing trials in which the participant reported the subjective experience of a color). Indeed, synesthetes’ “no color” trials are sufficient to classify synesthesia with above-chance accuracy (DeLong Test, $\mathrm{AUC}=0.76, z=5.11,p<0.001,\text{ AUC} 95\% \mathrm{CI} [0.66, 0.86]$); at the ideal threshold (maximizing sensitivity and specificity), sensitivity was 62.5% and specificity was 85.4%.

In sum, synesthetes behave measurably differently from non-synesthetes even when choosing colors for letters for which they report not having a conscious experience of color. By forcing all participants to choose a color for every trial, this phenomenon can be measured and explored.

## Discussion

Two types of “skipped” trials are potentially important influences on synesthesia experiments. One type of skipped trial might be termed a “low effort” trial: participants “skip” a trial if they do not move the color picker from its randomly initialized value. Most participants skip at least one trial, and a small subset of participants (~10% of self-reported non-synesthetes) skip at least 20% of all trials. This is a source of noise in grapheme–color association data that is relatively simple to account for: SynesthesiaColorPicker records the random starting value, so it is possible to exclude trials or participants based on whether the color picker was moved.

An alternative way to address “skipped” trials might be to force participants to move the color picker before they are allowed to proceed to the next trial (Cluskey et al., 2019). For researchers who prefer to implement this, it is trivial to turn on “Force Response” in Qualtrics, which will hide the “next” arrow until the participant interacts with the color picker. However, this introduces a confound in the “random” responses of participants, because disengaged participants who answer randomly (e.g., moving the color picker as little as possible, or clicking a completely random point) may choose colors differently from engaged participants who answer randomly (e.g., they may choose a more “pure” or “named” color, or draw from some other inhomogeneous distribution or “palette”; Rouw & Root, 2019). Furthermore, this makes it considerably more difficult to detect participants who are truly disengaged from the task. For example, approximately 5% of non-synesthete participants skip more than 60% of all trials (Fig. 3). It seems prudent to exclude these participants, who are clearly disengaged, but such behavior would be difficult to reliably detect if participants were forced to move the color picker on every trial (disengaged participants would be indistinguishable from engaged participants with inconsistent color associations). For this reason, SynesthesiaColorPicker does not force participants to move the color picker from its randomly initialized value, with the expectation that some trials or participants will be later excluded from the analysis.

One important caveat is that not all “skipped” trials are necessarily genuinely “skipped”: participants may have left the color picker at its random starting value because it was coincidentally close to the color that they were intending to choose. For this reason, I chose to exclude participants rather than trials. It is not trivial to determine the optimal proportion of skipped trials above which a participant should be excluded. In Root and Rouw (2025), we used a very conservative threshold (participants who skip 50% or more trials surely do so due to lack of effort, rather than coincidental alignment with color choices). In the future, a more optimal threshold might be derived via simulation studies (i.e., to determine the expected number of skipped trials as a function of test–retest consistency, color choices, and color space geometry), or via more complicated analyses (e.g., comparing the color category of a skipped trial to the color category of the same letter at retest).

The other type of “skipped” trials are “no color” trials for synesthetes: experimenters sometimes allow synesthetes to report that they do not experience a color, at which point the color picker question for that trial is skipped (they are not forced to choose a color). In the SynesthesiaColorPicker consistency test, synesthetes reported whether or not they experienced a color for each letter, but then were forced to choose a color regardless of their answer on the subjective questionnaire. Intriguingly, self-reported synesthetes’ “no color” trials are still significantly more consistent than the color associations of self-reported non-synesthetes. This raises not only technical and methodological questions, but also a more fundamental question about the nature of these consistent-yet-unconscious grapheme–color associations in synesthetes. For these reasons, in the SynesthesiaColorPicker test–retest consistency templates, the report of conscious experience (no color, one color, or multiple colors) is separated from the color picker task, and participants must answer both questions for each letter. This allows for future studies to explore this phenomenon, hopefully yielding new insights into the relationship between consistency and the conscious experience of synesthesia.

## General discussion

The present work introduces SynesthesiaColorPicker, an open-source, mobile-friendly color picker tool designed to standardize and improve measurement of color associations in synesthesia research. In two empirical studies, subtle design features in color picker tools were shown to significantly influence the data collected from both synesthetes and non-synesthete controls. Study 1 demonstrates that the linear hue slider with fixed endpoints used in the Synesthesia Battery led to an overrepresentation of colors corresponding to those endpoints. By contrast, the circular hue wheel in SynesthesiaColorPicker eliminated this bias, as it lacks endpoints and randomizes the orientation of the hue spectrum. This design ensures that no specific hues are inadvertently emphasized, leading to a more uniform distribution of color selections that better reflect participants' genuine associations. Study 2 demonstrates that a notable proportion of non-synesthete controls frequently skipped trials, potentially biasing consistency measures and affecting the sensitivity and specificity of synesthesia diagnostics. Furthermore, when synesthetes were required to select a color even for letters they reported as having “no color,” their responses were still significantly more consistent than those of non-synesthetes, suggesting that synesthetes might possess underlying consistent associations even when not consciously aware of them (an intriguing question for future studies). By mandating a color selection on every trial and by recording initial color values, SynesthesiaColorPicker allows researchers to account for and analyze these nuances in participant responses.

These studies underscore the importance of subtle methodological considerations in synesthesia research. Some methodological choices—for example, in how color associations are elicited and recorded—can inadvertently obscure genuine differences between synesthetes and non-synesthetes or can suggest differences when none are present. Furthermore, when methodological choices vary between experiments, it is difficult to know whether to ascribe differences in results to participants’ experiences or to the influence of the methodological choices. SynesthesiaColorPicker addresses these issues by offering a standardized platform that minimizes potential biases. Its open-source nature and compatibility with popular online experiment platforms further lower the barrier to entry for researchers, promoting more consistent and comparable findings across studies.

The extensibility of SynesthesiaColorPicker (it is a “plugin” that can be combined with other tools) allows it to be modified or extended to improve measurement of associations even further. The “Painter” demo on GitHub illustrates one example: SynesthesiaColorPicker can be combined with an HTML5 Canvas-based drawing application to allow synesthetes to “paint” more complex multicolored associations. SynesthesiaColorPicker could also be used as a starting point to design a more complex tool to capture additional dimensions of synesthetic experience, such as the texture or spatial qualities of color associations. SynesthesiaColorPicker is published under a GNU GPL License, so users are welcome to modify it in any way they see fit and publish these modifications (with attribution to the original, by citing this article).

In conclusion, SynesthesiaColorPicker represents a significant advancement in the methodological toolkit available to synesthesia researchers. It is free to use and includes templates allowing researchers with no coding experience to easily customize their own color picker experiment, lowering the barrier to entry for synesthesia research. In addition, by eliminating design-induced biases and accommodating nuanced participant responses, SynesthesiaColorPicker enhances the validity of data on synesthetic color associations. Furthermore, its open-source design allows researchers with more coding experience to use it as a starting point to design more complex experiments. I hope that the community finds SynesthesiaColorPicker helpful, and I look forward to seeing how it is used in future studies.

## Data Availability

All data and code to reproduce the figures and results can be downloaded from OSF: https://osf.io/vdfm7/. Experimental materials (SynesthesiaColorPicker) are on GitHub: https://github.com/nicholasbroot/synesthesiacolorpicker.

## Footnotes

1 In the newest version of the Synesthesia Battery, endpoints are now random; this concerns data collected with the battery from its conception until approximately 2020.
